# The REVIVE Project: From Survival to Holistic Recovery—A Prospective Multicentric Evaluation of Cognitive, Emotional, and Quality-of-Life Outcomes in Out-of-Hospital Cardiac Arrest Survivors

**DOI:** 10.3390/jcm14113631

**Published:** 2025-05-22

**Authors:** Alice Mandrini, Marco Mion, Roberto Primi, Sara Bendotti, Alessia Currao, Leila Ulmanova, Carlo Arnò, Filippo Dossi, Cristian Fava, Daniele Ghiraldin, Davide Pegorin, Paola Genoni, Diego Maffeo, Cinzia Dossena, Silvia Affinito, Giovanni Bertazzoli, Francesco Cipullo, Cecilia Fantoni, Matteo Della Torre, Silvia Frattini, Gioele Papi, Angelica Praderio, Luca Tarantino, Simone Savastano, Enrico Baldi, all the LombardiaCARe Researchers

**Affiliations:** 1 Division of Cardiology, Fondazione IRCCS Policlinico San Matteo, 27100 Pavia, Italy; a.mandrini@smatteo.pv.it (A.M.); m.mion@smatteo.pv.it (M.M.); r.primi@smatteo.pv.it (R.P.); s.bendotti@smatteo.pv.it (S.B.); a.currao@smatteo.pv.it (A.C.); l.ulmanova@smatteo.pv.it (L.U.); s.savastano@smatteo.pv.it (S.S.); 2 Cardiac Arrest and Resuscitation Research Team (RESTART), Fondazione IRCCS Policlinico San Matteo, 27100 Pavia, Italy; 3 Anglia Ruskin School of Medicine & MTRC, Chelmsford CB1 1PT, UK; 4 Department of Public Health, Experimental and Forensic Medicine, University of Pavia, 27100 Pavia, Italy; 5 Department of Internal Medicine and Medical Therapy, University of Pavia, 27100 Pavia, Italy; 6 Ospedale di Circolo e Fondazione Macchi di Varese, ASST Sette Laghi, 21100 Varese, Italy; carlo.arno95@gmail.com (C.A.); filippo.dossi@asst-settelaghi.it (F.D.); 7 Ospedale Carlo Poma, ASST di Mantova, 46100 Mantova, Italy; cristian.fava@asst-mantova.it; 8 Spedali Civili, 25123 Brescia, Italypegorin.davide@gmail.com (D.P.); 9 Ospedali Sant’Anna di Como e Sant’Antonio Abate di Cantù, ASST Lariana, 22063 Cantù, Italy; paola.genoni91@gmail.com; 10 Poliambulanza, 25124 Brescia, Italy; diego.maffeo@poliambulanza.it; 11 Ospedale Maggior di Crema, ASST di Crema, 26013 Crema, Italy; cinzia.dossena@asst-crema.it; 12 Ospedale di Legnano, ASST Ovest Milanese, 20025 Legnano, Italy; silviaaffinito@gmail.com; 13 Ospedale Maggiore di Lodi, ASST Lodi, 26900 Lodi, Italy; giovanni.bertazzoli@asst-lodi.it (G.B.); francesco.cipullo97@gmail.com (F.C.); 14 Ospedale Humanitas Mater Domini, 21053 Castellanza, Italy; cecilia.fantoni@materdomini.it; 15 Ospedale di Cremona, ASST Cremona, 26100 Cremona, Italy; matteo.dellatorre@asst-cremona.it (M.D.T.); silvia.frattini@asst-cremona.it (S.F.); gioele.papi@asst-cremona.it (G.P.); 16 Ospedale di Manerbio, ASST Garda, 25025 Manerbio, Italy; angelica.praderio@asst-garda.it; 17 Ospedale di Chiari, ASST Franciacorta, 25032 Chiari, Italy; luca.tarantino@asst-franciacorta.it

**Keywords:** out-of-hospital cardiac arrest, health status assessment, patient outcome assessment, cognitive dysfunction, quality of life, emotional distress, survivorship

## Abstract

**Background/Objectives**: Most survivors of out-of-hospital cardiac arrest (OHCA) may suffer from cognitive, mental difficulties, and fatigue, which negatively impact their quality of life, despite a good physical recovery. However, no definitive data are available on this topic, so this study aims to assess the feasibility and acceptability of a centralized, sub-regional screening system for OHCA survivors in Italy and the prevalence of these disorders. **Methods**: OHCA survivors discharged with good neurological outcomes (Cerebral Performance Category (CPC) ≤ 2 and modified Ranking Scale (mRS) ≤ 3) from hospitals in the “Lombardia CARe” registry will be evaluated by a clinical psychologist using the Montreal Cognitive Assessment (MoCA), Hospital Anxiety and Depression Scale (HADS), EQ-5D-5L for quality of life, and the Impact of Event Scale-Revised (IES-R) at pre-discharge or within 15 days and then at 1, 3, 6, and 12 months. Patients with clinical issues will be referred for psychological support or to a community rehabilitation program. Feasibility will be defined as a recruitment rate ≥ 80% and acceptability as a retention rate ≥ 50% over 12 months. **Results**: Based on historical data from the Lombardia CARe, an estimated 350 eligible survivors are expected, which will allow estimation of a prevalence ranging between 20% and 30% with 5% precision and 95% confidence. **Conclusions**: This study will be the first in Italy to evaluate the feasibility and acceptability of a centralized, sub-regional system for pre-/post-discharge evaluation of cognitive impairment, mental health, and quality of life in a large cohort of OHCA survivors, documenting the prevalence of these disorders.

## 1. Introduction

Out-of-hospital cardiac arrest (OHCA) is a leading cause of mortality worldwide, with overall survival rates estimated at 5–10% [1,2]. In Italy, about 60,000 OHCAs occur each year, with an incidence of about 1 case per 1000 inhabitants [3]. Among those who do survive, many achieve a good physical recovery yet may suffer from pronounced emotional or cognitive sequelae (e.g., rates of emotional problems and cognitive impairment at 6 months have been estimated at around 15–30% and 40–50%, respectively), along with fatigue [4,5,6]. These challenges can hinder a return to everyday life for both patients and relatives [7,8,9].

Guidelines from the European Resuscitation Council on the treatment of OHCA emphasize the importance of a systematic follow-up to screen for cognitive and emotional problems—for example, development of anxiety and depression or post-traumatic stress disorder symptoms—and to provide appropriate psychological support for patients and their families [6]. The importance of identifying (and meeting) the care and rehabilitation needs of survivors and their family members has also been recently highlighted by the Resuscitation Council UK [10] and translated for adoption by Fondazione IRC—the Italian non-profit organization dedicated to advancing the science and practice of resuscitation [11]. Some examples of post-discharge follow-up systems have been reported [12,13] alongside a framework for a multidisciplinary, guideline-based approach to improve the cardiac arrest pathway [14]. Guidelines and consensus statements also encourage the creation of multidisciplinary, holistic pathways of assessments and care [15,16,17]. Despite that, few structured programs exist globally, and few are currently in place in Italy. It is currently unclear which framework is best suited to deliver a comprehensive follow-up service that caters to the psychological/cognitive and physical needs of these patients; in addition, each healthcare system has unique implementation challenges. That notwithstanding, resource availability (i.e., personnel able to deliver the service) and access to appropriate training (i.e., how to deliver the service) are likely to be key issues.

During 2017–2018, the Cardiology Division of the Fondazione IRCCS Policlinico San Matteo, which is the coordinating centre of the Lombardia Cardiac Arrest Registry (LombardiaCARe), in collaboration with a non-profit organization (Pavia nel Cuore ODV) and the Psychiatry Unit of the University of Pavia, promoted a pilot study on the assessment of the psychological and emotional state of out-of-hospital cardiac arrest survivors in the Province of Pavia. This project showed that, in line with international data, more than one-third of patients discharged with a good neurological outcome (mRS ≤ 3) showed symptoms of at least moderate depression [18]. The incidence of depression was also higher in younger patients (<50 years old), in line with other observational studies [19,20,21], with mental health being particularly affected. The health-economic implications of this long-term burden are likely significant.

Building on those pilot data, this study aims to (a) investigate the feasibility and acceptability of a centralized, sub-regional assessment and follow-up system; and (b) to investigate the prevalence of psychological distress, cognitive impairment, and fatigue, as well as the degree of health-related quality of life in a large, multicentre cohort of OHCA survivors.

## 2. Materials and Methods

### 2.1. Study Design

This is a prospective, multicenter observational protocol, spanning 24 months, across 22 hospitals affiliated with the Lombardy Cardiac Arrest Registry (“Lombardia CARe”—a full list of the hospitals is provided in Table 1). This network covers a population of approximately 4.3 million people and includes densely populated urban areas and sparsely populated rural and mountainous locations.

**Figure 1:**
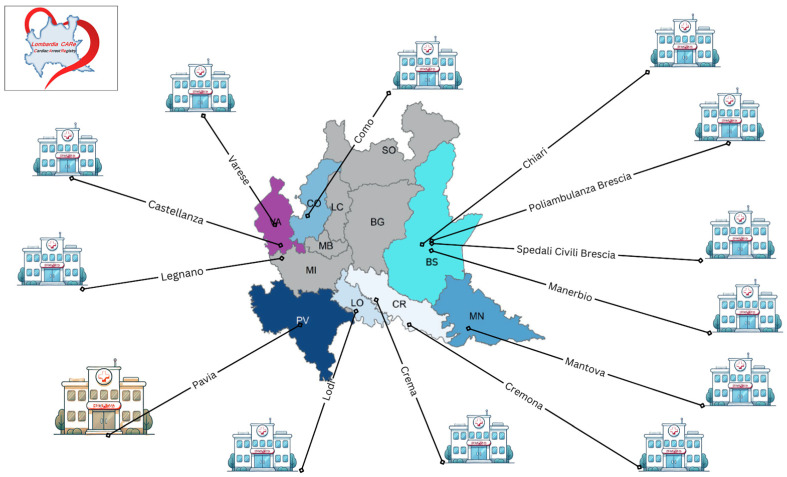
Location of the recruiting hospitals in the Lombardy region. Different colors identify the different provinces.

In the REVIVE project, each of the 22 hospitals will have a named contact person (usually a senior cardiologist) who will enroll eligible survivors and inform the study team of the clinical status and readiness to be assessed before hospital discharge. Based on registry data from 2022, >90% of patients with a good neurological outcome (CPC 1–2; mRS ≤ 3) were discharged from 11 hospitals affiliated to this network, therefore these sites have been highlighted in Table 1 as ‘Main Recruiters’ and a picture describing their approximate location in the Lombardy region is provided (Figure 1).

The REVIVE project plans to enroll consecutive OHCA survivors meeting eligibility criteria and to follow them up for 12 months post-discharge. Survivors will be assessed at five different timepoints: **T0**—before discharge (or within 15 days post-discharge); **T1**: 1 month post-discharge; **T2**: 3 months post-discharge; **T3**: 6 months post-discharge; **T4**: 12 months post-discharge.

At each time point, the same battery of questionnaires will be administered **in-person or over the telephone/via telemedicine**, based on patient preference and feasibility.

Should a significant need be identified, the study team will, in the first instance, consider whether psychological therapy/cognitive rehabilitation can be provided in-house; however, it is expected that most patients will be referred to community-based rehabilitation programmes or other specialists where required.

#### 2.1.1. Inclusion Criteria

Patient must be ≥18 years and have survived an OHCA with attempted resuscitation by the Emergency Medical System. They display a **CPC 1–2 or mRS ≤ 3** at discharge and are capable of providing written informed consent.

#### 2.1.2. Exclusion Criteria

Patients unable to speak the language to a sufficient degree to be able to participate in the assessments will not be recruited. In addition, patients with acute, currently unresolved psychological disorders of moderate/severe intensity—e.g., major depression, schizophrenia—will not be recruited in this trial.

#### 2.1.3. Enrolment and Assessments

The Lombardia CARe Registry records all admissions of patients who have suffered an out-of-hospital cardiac arrest across seven provinces in the Lombardy region, updated to the previous 24 h. This registry is monitored daily by the team involved in this study; for patients admitted to the IRCCS Policlinico San Matteo Hospital in Pavia, their clinical history (including survival and neurological status) is monitored via the hospital internal electronic system and, if eligible, they are approached to provide consent for participation to this study close to their discharge day, usually in the cardiology ward. For those patients admitted to one of the ten other main recruiting hospitals (Figure 1), the local investigator will approach them for screening and enrollment. They will also inform the study team about their clinical status and readiness for assessment before hospital discharge. To minimize the risk of patients being missed, an email will be sent to all the investigators of the main recruiting hospitals every Monday and every Friday. The secondary recruiting centres do not have an allocated local investigator, so they will be contacted as and when required.

Most eligible patients are supposed to be approached before hospital discharge; if not, the study management team will contact them within 15 days post-discharge. Following informed consent, baseline assessment will be conducted by a clinical psychologist, either in-person (for patient admitted to Fondazione IRCSS San Matteo hospital, Pavia) or remotely (via telephone call or telemedicine, for all other patients), using measures of cognitive ability (Montreal Cognitive Assessment [22]), anxiety and depression [23], health-related quality of life [24], and symptoms of post-traumatic stress disorder [25]. Parallel versions of the cognitive assessment will be administered to reduce practice effects. A list of all measures and their purpose is described in Table 2.

In addition to quantitative instruments, starting from the first follow-up after hospital discharge (that is, the 1-month follow-up), patients will also undergo a brief semi-structured, qualitative interview focused on their own subjective experience of returning to day-to-day life, fatigue, cognitive problems, mood difficulties, and their ability to access appropriate information/support about their condition (Appendix A; Table A1).

### 2.2. Criteria for Feasibility and Acceptability

Feasibility will be defined quantitatively by examining the **recruitment rate** (i.e., the proportion of eligible patients who consent to participate) and the **retention rate** (i.e., the proportion of enrolled patients who complete scheduled follow-up assessments over the 12-month period). In the absence of literature benchmarks and prior pilot data, a pragmatic threshold *of 80% recruitment rate* of eligible patients will be set as a measure of feasibility.

Acceptability will be operationalized as an *overall retention rate of 50% or more of the recruited patients* over 12 months. In addition, this study will look at feasibility and acceptability by follow-up modality, comparing the recruitment and attrition rates between face-to-face assessments at the main hospital and remote assessments at the other hospitals. A significantly lower recruitment rate or higher attrition rate in the remote modality, as compared to the face-to-face approach, will be interpreted as indicative of reduced feasibility and acceptability.

### 2.3. Data Management

All data will be pseudo-anonymized and stored on a secure Research Electronic Data Capture (REDCap^®^, Vanderbilt University, Nashville, TN, USA) platform hosted by the Fondazione IRCCS Policlinico San Matteo in Pavia. Only authorized study staff will have access. Data collected will include demographic data (age, sex, etc.); OHCA- and resuscitation-related variables (time to ROSC, initial rhythm, neurological outcome); scores from MoCA, HADS, EQ-5D-5L, and IES-R at each time point, and qualitative information collected in the semi-standardized follow-up interviews.

### 2.4. Statistical Methods

Baseline characteristics will be reported as means ± SD, or medians [IQR] for continuous variables, and as frequencies (%) for categorical variables. Prevalence of psychological distress, cognitive impairment, fatigue, symptoms of post-traumatic stress, and reduced quality of life will be estimated at all timepoints; exact 95% confidence intervals will be calculated. Reduced quality of life will be defined as an EQ-5D-5L VAS score at least 7 points lower than the age-matched Italian general population mean [26], based on established minimal clinically important differences identified in chronic disease populations [27,28].

Incidence rates (expressed per 100 person-years) for mood disorders, post-traumatic stress symptoms, reduced quality of life, and cognitive impairment will be calculated for participants free of the respective condition at baseline. Incident cases will be defined as follows: a HADS subscale score > 8 for mood disorders (either anxiety or depression subscale), IES-R ≥ 33 for post-traumatic stress symptoms, a MoCA score < 26 for cognitive impairment, and a decrease of ≥7 points from the age-matched normative mean on the EQ-5D-5L VAS for reduced quality of life. Baseline status will be determined using the initial T0 assessment; if T0 data are missing, the T1 (1-month) assessment will be used. Participants with missing data at both T0 and T1 for a given outcome will be excluded from the incidence rate calculation for that outcome. Incidence rates will be reported with exact 95% confidence intervals.

Longitudinal changes in HADS, MoCA, EQ-5D-5L, and IES-R from T0 to T4, as well as differences in the retention rate between patients seen remotely or face-to-face, will be analyzed using mixed-effects models (linear or logistic) incorporating time and potential covariates (e.g., age, sex, comorbidities). Models will initially include random intercepts and slopes at participant and hospital levels. Different residual covariance structures (e.g., first-order autoregressive [AR(1)]) will also be explored. Model selection will be guided by the Akaike Information Criterion (AIC) and Bayesian Information Criterion (BIC); simpler random intercept-only models will be adopted if convergence issues arise. Missing data will be handled using Multiple Imputation by Chained Equations (MICE), assuming a Missing At Random (MAR) mechanism. Twenty imputed datasets will be generated, incorporating all key predictors and outcomes, as well as hospital ID. Participants who completely withdraw from this study will be treated as censored at their last observation without further imputation. Sensitivity analyses comparing complete-case and imputed datasets will be conducted to assess the robustness of the findings. A *p*-value < 0.05 (two-sided) will denote statistical significance.

Qualitative data will be derived from detailed notes taken during semi-structured interviews; the analysis will employ a framework approach, well-suited for applied qualitative research with pre-defined areas of inquiry [29]. The analytic process will involve familiarization with the notes, followed by the development of a thematic framework based on the predetermined domains. Data will then be indexed using both deductive codes—stemming from the interview guide—and emergent codes that may arise from the content of the notes. A matrix will be constructed to chart responses across cases, enabling systematic cross-case comparisons.

## 3. Expected Results

### 3.1. Investigation of the Feasibility and Acceptability of a Sub-Regional “Hub and Spoke” Model of Post-Discharge Care Delivery

There is growing evidence that management of OHCA in “cardiac arrest centres”, and regionalization of post-cardiac arrest care may be beneficial to improve outcomes ranging from survival to long-term functional abilities (see [30] for a review)—even though there is still no definitive evidence in this area. The feasibility and acceptability of providing post-discharge follow-up care in a centralized manner at a regional/sub-regional level, however, are still unexplored.

Potential benefits of this approach revolve around leveraging the infrastructure, resources, personnel, and experience of a leading high-volume “cardiac arrest centre” to provide high-quality post-OHCA care in line with international guidelines; predictable barriers, however, could be encountered in the acceptability of this service when provided remotely, especially for older people and for those with neurocognitive impairments and/or psychological difficulties. It is expected that REVIVE will help to provide novel evidence in this area to inform the development of new guidelines.

### 3.2. Identification of Prevalence of Mood Disorders in OHCA Survivors

Based on Lombardia CARe figures, approximately 5200 OHCAs occur yearly in the regional area, and, in about 3500 of them, resuscitation is attempted. Of these, about 600 survive to hospital admission, and about 175 per year are discharged alive with CPC 1–2 or mRS ≤ 3. Therefore, over 24 months, about 350 eligible survivors are expected. Assuming 80% of them consent to be included in this study (about 280), this cohort will be sufficient to identify the presence of symptoms of at least mild anxiety or depression in 25% of cardiac arrest survivors at 6 months from the event, with 5% precision and 95% confidence (Appendix A, Table A2). Although the prevalence of mood disorders after OHCA has been investigated by several studies, REVIVE will be the first one to do so at the sub-regional level in Italy.

## 4. Discussion

Survivors of OHCA face a broad array of challenges, extending beyond the immediate physical aftermath to include psychological distress, cognitive impairments, and chronic fatigue. Depression is notably associated with higher mortality in this population [31]. Despite increased awareness, however, few structured pathways exist to identify and address these non-physical sequelae. Current pathways involve follow-ups being completed mostly or entirely in person, by the hospital that admitted the patients following their OHCA [12,14]; despite this approach having clear advantages (namely, full access to the clinical history of the patient; patient being already familiar with the hospital/treating team; face-to-face meeting being more accessible to some patients, etc.), it also requires more resources in terms of personnel, training required and clinic space.

The prospective, multicentric REVIVE project aims to investigate the feasibility and acceptability of a centralized, sub-regional system to investigate mood, cognition, fatigue, psychological wellbeing, and overall quality of life at multiple time points up to 12 months post-discharge. It also aims to be the first study to systematically assess psychological distress, cognitive status, and quality of life in a sizable Italian cohort of OHCA survivors.

Study limitations may include attrition (e.g., among patients with cognitive deficits or significant psychological difficulties) and the non-randomized nature of the intervention. Remote/telemedicine follow-up options will aim to reduce dropout; however, they might also constitute a barrier for some patients who feel less comfortable using new technology.

Should the REVIVE study demonstrate the need for a tailored psychological approach and that our approach is feasible and welcomed by the patients, it could serve as a guide to build up similar and permanent programs to help patients thrive and not simply survive after a cardiac arrest.

The results are expected to guide future recommendations on the delivery of follow-up/assessment services and help establish new standards of post-resuscitation care.

## 5. Conclusions

The “REVIVE Project: from survival to holistic recovery” will provide comprehensive insights into the long-term psychological, cognitive, and physical sequelae experienced by OHCA survivors in a large Italian cohort, offering a robust foundation for optimizing follow-up care pathways and informing future guidelines. By leveraging a centralized, sub-regional assessment model, our findings are expected to advance patient-centered care strategies and contribute significantly to establishing new standards in post-resuscitation management.

## Figures and Tables

**Table 1:** Hospitals participating in the *Lombardia CARe Cardiac Arrest Registry*, categorized into *Main Recruiters* and *Secondary Recruiters* in the REVIVE study.

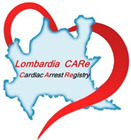	
Main Recruiters	Secondary Recruiters
**Fondazione IRCCS Policlinico San Matteo, Pavia**	ASST Valle Olona—Ospedale di Gallarate, Gallarate
**ASST dei Sette Laghi** **Ospedale di Circolo e Fondazione Macchi, Varese**	ASST di Mantova—Ospedale di Asola, Castiglione delle Stiviere
ASST di Mantova—Ospedale Carlo Poma, Mantova	Ospedale Sacra Famiglia Fatebenefratelli, Erba
ASST Spedali Civili di Brescia—Presidio Spedali Civili, Brescia	ASST di Lecco—Ospedale Alessandro Manzoni, Lecco
**ASST Lariana—Ospedale Sant’Anna, Como**	Ospedale Valduce, Como
**ASST di Lodi—Ospedale Maggiore, Lodi**	Ospedale Moriggia-Pelascini, Gravedona
**Fondazione Poliambulanza Istituto Ospedaliero, Brescia**	ASST Papa Giovanni XXIII—Ospedale Papa Giovanni XXIII, Bergamo
**ASST Ovest Milanese—Ospedale Civile, Legnano**	ASST Valle Olona—Ospedale di Busto Arsizio, Busto Arsizio
**ASST di Crema—Ospedale Maggiore, Crema**	ASST dei Sette Laghi—Ospedale di Cittiglio, Cittiglio
ASST di Cremona—Ospedale di Cremona, Cremona	ASST Valtellina e Alto Lario—Ospedale Civile, Sondrio
Humanitas Mater Domini, Castellanza	ASST Lariana—Ospedale Sant’Antonio Abate, Cantù
**Ospedale di Manerbio, ASST Garda, Manerbio, Brescia, Italy**	ASST dei Sette Laghi—Ospedale di Tradate, Tradate
**Ospedale di Chiari, ASST Franciacorta, Chiari, Brescia, Italy**	ASST di Pavia—Ospedale Civile, Vigevano
	Ospedale Erba-Renaldi, Menaggio
	Humanitas Research Hospital, Milano
	ASST di Pavia—Ospedale di Varzi, Varzi
	ASST Melegnano e Martesana—Ospedale di Melegnano, Melegnano
	ASST di Mantova—Ospedale di Pieve di Coriano, Pieve di Coriano
	ASST di Pavia—Ospedale Civile, Voghera

**Table 2:** Key assessment tools used for evaluating cognitive function, psychological distress, and health-related quality of life.

Assessment Tool	Purpose
Montreal Cognitive Assessment (MoCA)	Screens for cognitive deficits in memory, executive functions, visuospatial abilities, attention, language, and orientation.
Hospital Anxiety and Depression Scale (HADS)	Screens for anxiety and depression (7 items each). A subscale score > 8 suggests clinically relevant symptoms.
EuroQol-5 Dimensions-5 Levels (EQ-5D-5L)	Assesses health-related quality of life across five dimensions (mobility, self-care, usual activities, pain/discomfort, anxiety/depression), each on five severity levels. Includes a visual analogue scale (EQ VAS) indicating overall perceived health.
Impact of Event Scale-Revised (IES-R)	Assesses post-traumatic stress symptoms related to a life-threatening event (e.g., OHCA). A total score ≥ 33 frequently indicates clinically significant distress.

## Data Availability

The data of this study will be available upon reasonable request to the corresponding author.

## Abbreviations

The following abbreviations are used in this manuscript:

OHCA	Out-of-Hospital Cardiac Arrest
CPC	Cerebral Performance Category
mRS	modified Rankin Scale
MoCa	Montreal Cognitive Assessment
HADS	Hospital Anxiety and Depression Scale
IES-R	Impact of Event Scale-Revised
EQ-5D-5L	EuroQol-5 Dimensions-5 Levels
IRC	Italian Resuscitation Council
T0, T1, T2, T3, T4	Time0, Time1, Time2, Time3, Time4
ROSC	Return of Spontaneous Circulation

## Appendix A

**Table A1:** Semi-structured clinical interview for post-cardiac arrest survivors.

Semi-Structured Clinical Interview (English Translation)
**Cognition and Cognitive Functions:** *“Have you noticed any changes in your memory, ability to concentrate, or finding the right words after being discharged? How have these changes affected your daily activities?”* 2. **Emotional Well-Being and Mood:** *“In the past few weeks, have you experienced feelings of anxiety, low mood, or unwanted memories related to the cardiac arrest? How have these feelings impacted you or your family members?”* 3. **Fatigue and Physical Recovery:** *“Do you experience persistent fatigue, mobility difficulties, or physical limitations that make daily activities more challenging compared to before the cardiac arrest?”* 4. **Daily Life, Work, and Independence:** *“Have you encountered difficulties in resuming important daily activities, such as driving, managing household tasks, or returning to work? Do you feel you have the necessary support to face these challenges?”* 5. **Information Needs and Support Networks:** *“Do you and your family feel you have the necessary information and support—for example, understanding medication use, knowing what to expect in the coming months, and accessing emotional or peer support—to manage this phase of recovery effectively?”*
**Intervista clinica semi-strutturata (original italian version)**
**Cognizione e funzioni cognitive:** *“Ha notato cambiamenti nella sua memoria, nella capacità di concentrarsi o nel trovare le parole giuste dopo la dimissione, e in che modo questi cambiamenti influenzano le sue attività quotidiane?”* 2. **Cognizione e funzioni cognitive:** *“Ha notato cambiamenti nella sua memoria, nella capacità di concentrarsi o nel trovare le parole giuste dopo la dimissione, e in che modo questi cambiamenti influenzano le sue attività quotidiane?”* 3. **Benessere emotivo e umore:** *“Nelle ultime settimane, ha provato sentimenti di ansia, umore depresso o ricordi indesiderati legati all’arresto cardiaco? In che modo questi sentimenti hanno influito su di lei o sui suoi familiari?”* 4. **Affaticamento e recupero fisico:** *“Prova stanchezza persistente, difficoltà di mobilità o limitazioni fisiche che rendono le attività di tutti i giorni più impegnative rispetto a prima dell’arresto cardiaco?”* 5. **Vita quotidiana, lavoro e indipendenza:** *“Ha riscontrato difficoltà nel riprendere le attività quotidiane importanti, come guidare, gestire i lavori domestici o tornare al lavoro? Ritiene di avere il supporto necessario per affrontare queste sfide?”* 6. **Informazioni necessarie e reti di supporto:** *“Lei e i suoi familiari sentite di avere le informazioni e il sostegno necessari—ad esempio, comprendete la funzione dei farmaci, sapete cosa aspettavi nei prossimi mesi, e siete in grado di accedere a supporto emotive/di mutuo aiuto—per gestire al meglio questa fase del recupero?”*

**Table A2:** Precision of the proportion estimate according to the number of patients enrolled and the proportion found.

Expected Proportion	Absolute Precision with 95% Confidence									
	0.01	0.02	0.03	0.04	0.05	0.06	0.07	0.08	0.09	0.10
**0.01**	381	96	43	24	16	11	8	6	5	4
**0.11**	3761	941	418	236	151	105	77	59	47	38
**0.21**	6373	1594	709	399	255	178	131	100	79	64
**0.31**	8217	2055	913	514	329	229	168	129	102	83
**0.41**	9293	2324	1033	581	372	259	190	146	115	93
**0.51**	9600	2400	1067	600	384	267	196	150	119	96
**0.61**	9139	2285	1016	572	366	254	187	143	113	92
**0.71**	7910	1978	879	495	317	220	162	124	98	80
**0.81**	5913	1479	657	370	237	165	121	93	73	60
**0.91**	3147	787	350	197	126	88	65	50	39	32
